# A Case of Identity

**DOI:** 10.4103/0973-1229.27616

**Published:** 2006

Tonight at a classical dance recital
symbolizing the eternal quest of the soul-force for God
I too raised for myself some questions
As I saw the dancer adopt body twisted
beautifully gymnastic postures
like a question mark put before me
- who am I?

Am I the inquisitive streetwalker listening at pavements
to snake charmers’ aphrodisiac calls,
watching indolently as the fast cycling rogue
whirling past knocks her off, squeezes her breasts,
makes off
and leaves me to pacify her smothered adolescence.

Or am I the automaton
That hangs on to footboards and breezes past in fast trains
every morning and evening
the only sensation left in me being in my hands
holding on for dear life, and in my lips
wolf whistling as I swirl past platforms.

Or just the nimble fingers
typing away at jet-speed,
taking down bosses’ notes, thumbing across wads of money,
hand shaking
with prospective customers.

Or just tongues, tongues and more tongues
with mounds and mounds of sugar and ghee and adulterated oils
pouring it all into unknowing ears
furthering business prospects
feeding everyone around in slow poison doses
and fattening on pure ghee, purity of expression, conscience.

So that I can decorate my drawing room with costly paintings and
upholstery
watch expensive dance recitals
and answer question marks once in a while.
Ajai R. Singh

